# A Retrospective Study of Lateral Antebrachial Cutaneous Nerve Neuropathy: Electrodiagnostic Findings and Etiologies in 49 Cases

**DOI:** 10.3390/neurolint16050086

**Published:** 2024-10-10

**Authors:** Vasudeva G. Iyer, Lisa B. E. Shields, Michael W. Daniels, Yi Ping Zhang, Christopher B. Shields

**Affiliations:** 1 Neurodiagnostic Center of Louisville, Louisville, KY 40245, USA; pavaiyer@gmail.com; 2 Norton Neuroscience Institute, Norton Healthcare, Louisville, KY 40202, USAyipingzhang50@gmail.com (Y.P.Z.); 3 Department of Bioinformatics & Biostatistics, University of Louisville, Louisville, KY 40202, USA; michael.daniels@louisville.edu

**Keywords:** neurology, lateral antebrachial cutaneous nerve, musculocutaneous nerve, biceps tendon repair, iatrogenic nerve injury, sensory nerve conduction study

## Abstract

Background: The lateral antebrachial cutaneous nerve (LACN) is the terminal sensory branch of the musculocutaneous nerve and is rarely entrapped or injured. This study describes the electrodiagnostic (EDX) findings and etiologies of LACN neuropathy. Methods: This is a review of 49 patients with pain and/or paresthesia of the forearm who underwent EDX studies. The diagnosis of LACN neuropathy was based on clinical and sensory conduction abnormalities. Results: The most common etiology of LACN neuropathy was iatrogenic injury in 30 (61.2%) patients, primarily due to biceps tendon repair at the elbow (11 [36.7%]) and phlebotomy (5 [16.7%]). Fifteen (30.6%) patients sustained a non-iatrogenic injury at the proximal forearm/elbow, consisting of six (60%) laceration injuries and five (33.3%) stretch injuries. Four (8.2%) patients comprised the “other” etiology category, including two mass lesions causing LACN compression. Pain, paresthesia, and/or numbness in the LACN distribution were reported in 33 (67.3%), 27 (55.1%), and 23 (46.9%) patients, respectively. Hypoesthesia was detected in 45 (91.8%) patients, and dysesthesia in 7 (14.3%). The sensory nerve action potentials (SNAPs) of the LACN on the symptomatic side were absent in 44 (89.8%) patients. Of the five patients whose SNAPs of the LACN were detected, all had a decreased amplitude, and two had increased sensory latency. Conclusions: The most common etiology for LACN neuropathy in this series was iatrogenic injury; repair of biceps tendon at the elbow was the most frequent provoking cause. Protection of the LACN during surgical procedures at the elbow and forearm is vital to prevent iatrogenic injury.

## 1. Introduction

**Figure 1:**
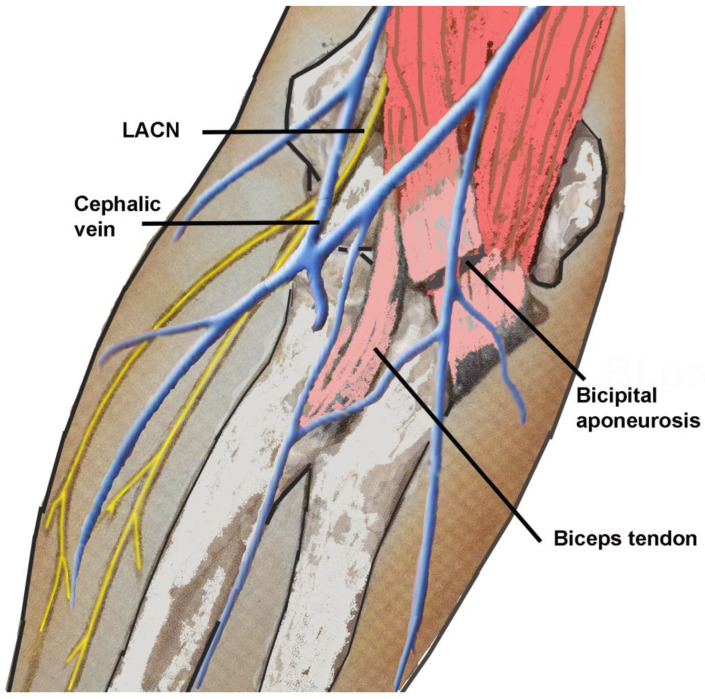
Anatomical proximity of lateral antebrachial cutaneous nerve to the biceps tendon, bicipital aponeurosis, and cephalic vein. LACN: lateral antebrachial cutaneous nerve.

The lateral antebrachial cutaneous nerve (LACN) is the distal sensory termination of the musculocutaneous nerve in the lateral aspect of the forearm [1,2,3,4,5]. After the musculocutaneous nerve gives off its muscular branches to the biceps brachii and brachialis muscles, it continues as the LACN. The LACN passes alongside the biceps brachii tendon and deep to the cephalic vein, after which it descends down the radial aspect of the forearm to the wrist where it is anterior to the radial artery. The LACN terminates at the base of the thenar eminence. The LACN is solely a sensory nerve and innervates the skin of the anterior half and radial posterior quarter of the forearm. Due to its anatomical proximity to the biceps brachii tendon, lateral epicondyle, antebrachial vein, and cephalic vein (Figure 1), the LACN is prone to iatrogenic injuries during repair of the biceps tendon [4,6,7,8,9,10], phlebotomy [4,11,12,13,14,15], prolonged use of retractors by surgical assistants [16], positional pressure during prolonged surgery [17], and steroid injection at the lateral epicondyle [18]. Entrapment neuropathy of the LACN may occur at the lateral margin of the biceps tendon/bicipital aponeurosis or at the site of emergence of the nerve through the deep fascia [1,2,3,4,5,16,19,20,21,22,23,24].

Dynamic compression at potential entrapment sites of the LACN during activities that require forcible elbow flexion/extension or forearm pronation has been postulated as an etiology of LACN neuropathy [1,2]. This condition has been associated with a variety of physical activities causing stretch injuries, occurring in high-level pitchers [25], throwing athletes [26], windsurfers [27], swimmers (backstroke) [28], tennis players (forceful overhead tennis stroke with the forearm pronated) [28], racquetball (backhand stroke) [28], restaurant servers from pressure on the lateral bicipital tendon by edges of heavy trays [29], boxers (following a punch in forceful elbow extension and forearm pronation) [30], basketball players (slam-dunking basketball and hanging on the rim) [28], repetitive forearm use while gardening [4], and photographers carrying a camera bag with the strap draped over the antecubital fossa for prolonged periods of time (“handbag paresthesia”) [31].

Patients with pain and paresthesia at the elbow and volar forearm should undergo a thorough sensory examination to document the topography of sensory deficit followed by electrodiagnostic (EDX) studies to confirm the LACN neuropathy. Characteristic findings of LACN neuropathy in the symptomatic forearm include sensory nerve action potentials (SNAPs) that are either absent, have a prolonged distal latency, or have a decreased amplitude compared to the asymptomatic side [2,18,29].

While there are many anecdotal reports of LACN neuropathy, there are few large series, leading to insufficient data regarding the causes of LACN neuropathy. The study of EDX-confirmed cases of LACN neuropathy is a valuable source of insight into this rare focal neuropathy of the upper extremity. In this report, the clinical and EDX findings of 49 patients with LACN neuropathy confirmed by EDX studies are described with discussion focusing on the etiology. The occurrence of iatrogenic injuries and the potential causes may provide insight into the development of precautionary measures.

## 2. Materials and Methods

### 2.1. Electrodiagnostic Studies

Under an Institutional Review Board (IRB)-approved protocol, we performed a 13-year (2 August 2010–22 June 2023) retrospective analysis of patients with pain and/or paresthesia of the forearm referred for EDX studies. Neurological examinations of the arms as well as nerve conduction velocity (NCV) and needle EMG studies were conducted. The EDX studies took place at our American Association of Neuromuscular & Electrodiagnostic Medicine (AANEM)-accredited location. The LACN nerve conduction studies (NCSs) were conducted according to the technique described by Buschbacher and colleagues [32].

Our Neurodiagnostic Center evaluates approximately 1000 patients annually who are referred for EDX studies by hand surgeons, neurosurgeons, orthopedic surgeons, neurologists, and internists. Of the patients referred to our Neurodiagnostic Center during the time period of this study, we retrospectively included all patients with LACN palsy confirmed by nerve conduction studies. All patients who were referred to our Neurodiagnostic Center were included over the specified time period of the study.

### 2.2. Inclusion and Exclusion Criteria

**Figure 2:**
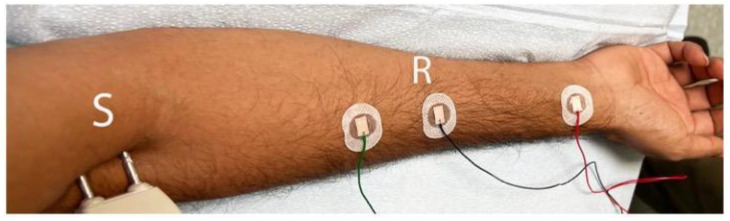
Nerve conduction study of lateral antebrachial cutaneous nerve showing positioning of the stimulating (S) and the recording electrodes (R) at 10 cm. The ground electrode is placed between the stimulating and recording electrodes. The reference is over the wrist.

Inclusion criteria were as follows: (1) sensory disturbance (pain/paresthesia/hypoesthesia/allodynia) in the lateral aspect of the forearm and (2) NCSs show absence of sensory nerve action potentials (SNAPs) over the LACN; peak-to-peak amplitude less than 50% of the unaffected side; or peak latency which is twice or more than that of the unaffected side. The nerve was stimulated at the distal upper arm just lateral to the biceps tendon (S), and the recording electrode (R) was placed 10 cm distally on a line to the radial pulse (Figure 2). A peak latency ≥ 2.6 ms and peak-to-peak amplitude of ≤3 μV were considered abnormal [32]. The asymptomatic side was studied for comparison. The nerves of the unaffected side were studied in all cases. Median, ulnar, superficial radial, and medial antebrachial cutaneous nerves were also studied along with the needle EMG of the upper extremity muscles to confirm or rule out brachial plexopathy/cervical radiculopathy. There were no exclusion criteria.

Several metrics were collected including the patients’ gender and age, laterality (left/right), symptom onset (acute/chronic), symptoms (pain/paresthesia/numbness) and signs (hypoesthesia/dysesthesia) in the distribution of the LACN, SNAP findings (absent/decreased amplitude/increased latency), and clues to the etiology of the LACN neuropathy.

### 2.3. Statistical Analysis

A retrospective observational study assessed the relationship between NCV SNAP levels and three key symptoms: pain, paresthesia, and numbness. The NCV SNAP variable was categorized into three groups: (1) Absent, (2) Decreased Amplitude, and (3) Decreased Amplitude and Increased Latency. Patients were divided based on the NCV SNAP group they belonged to, and comparisons were made for age, gender, and symptom outcomes.

Descriptive statistics were generated to summarize patient demographics and clinical characteristics across the NCV SNAP groups. The mean and standard deviation were calculated for continuous variables such as age, while categorical variables (e.g., gender, affected side) were presented as frequencies and percentages. Table 1 used chi-square tests for categorical comparisons, and a one-sided t-test was applied for the age variable. Due to the small sample sizes and non-normal data distribution in Table 2 and Table 3, non-parametric tests were employed. The Wilcoxon Rank Sum test was used for continuous variables, while Fisher’s exact test was used for categorical variables. Fisher’s exact test is appropriate for small sample sizes and contrasts observed frequencies against expected frequencies in contingency tables. A *p*-value of <0.05 was considered statistically significant. All analyses were performed using R software (version 4.4.1, R Core Team (2023). R: A Language and Environment for Statistical Computing. R Foundation for Statistical Computing, Vienna, Austria. https://www.R-project.org).

### 2.4. Institutional Review Board Approval of Research

Informed consent was obtained from all patients. The IRB determined that our study was exempt under 45 CFR 46.104(d)(4). The IRB number is 23-N0146. The IRB approval date was 25 July 2024.

## 3. Results

### 3.1. Demographics

A total of 49 patients were diagnosed with LACN neuropathy based on clinical and EDX findings (Table 1). The mean age was 48.4 years (range: 16–81 years), and the majority (31 [63.3%]) of patients were male. A one-sided t-test comparing the mean age to 50 years resulted in a *p*-value of 0.428, indicating no statistically significant difference from 50 years. The *p*-value for the gender distribution was 0.063, suggesting a marginal difference that was not statistically significant. The LACN neuropathy was more common on the right side (28 [57.1%]). The difference between sides was not statistically significant (*p* = 0.317). Forty-three (87.8%) patients were right-hand dominant, 3 (6.1%) were left-hand dominant, and 3 (6.1%) were ambidextrous. The *p*-value for the distribution of hand dominance was highly significant (*p* < 0.001), indicating a strong right-hand dominance in the cohort. The symptomatic side corresponded to hand dominance in 8 (57.1%) patients, which was not significantly different from the symptomatic side not corresponding to the dominant hand (*p* = 0.317). A total of 44 (89.8%) patients had an acute onset of symptoms, while 5 (10.2%) experienced a gradual onset. This difference was statistically significant (*p* < 0.001), indicating a clear predominance of acute onset cases.

**Figure 3:**
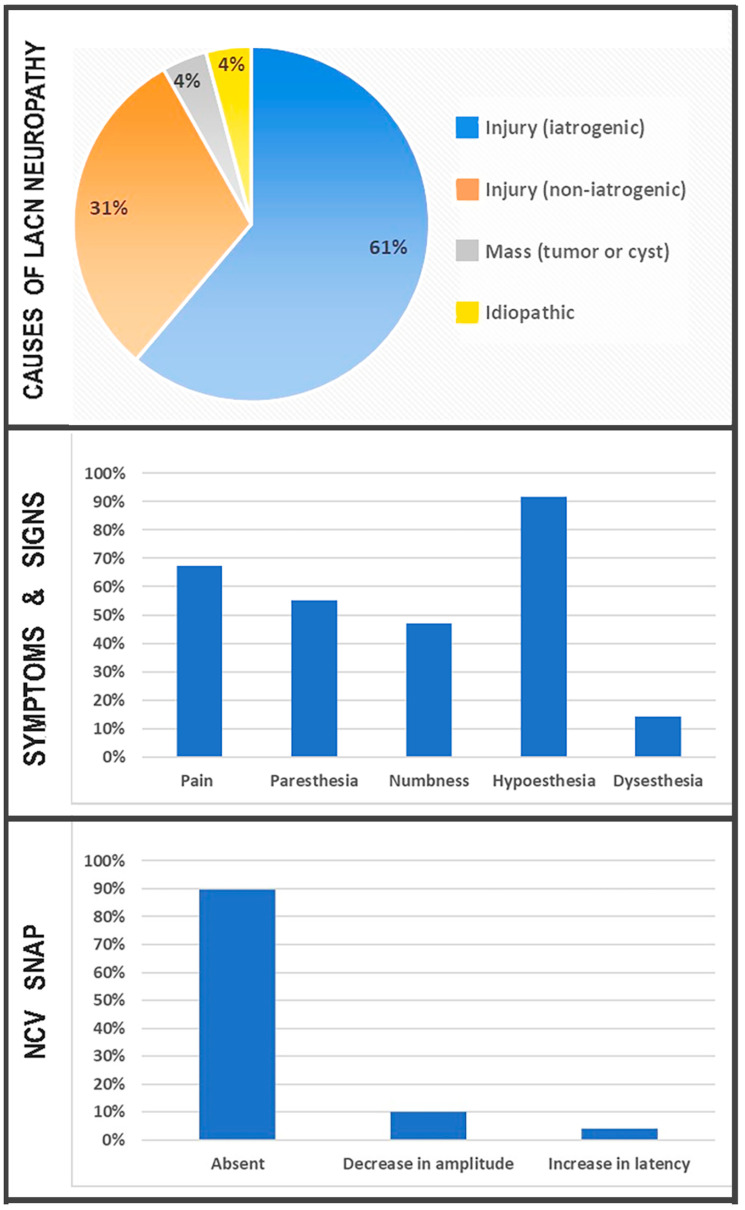
Causes, symptoms, signs, and NCV SNAP findings associated with LACN neuropathy. LACN: lateral anterior cutaneous nerve. NCV: nerve conduction velocity. SNAP: sensory nerve actions potentials.

Figure 3 summarizes the causes, symptoms, signs, and NCV SNAP findings associated with LACN neuropathy in our study.

### 3.2. Etiologies

The most common etiology of LACN neuropathy was iatrogenic injury in 30 (61.2%) patients, primarily due to biceps tendon repair at the elbow (11 [36.7%]) and phlebotomy (5 [16.7%]) (Table 1). Two patients underwent intense physical therapy several weeks following shoulder replacement surgery which created symptoms of LACN neuropathy. Fifteen (30.6%) patients experienced non-iatrogenic injury at the proximal forearm/elbow, with six (40.0%) sustaining a laceration injury and five (33.3%) incurring a stretch injury. Patients with stretch injuries gave a history of prolonged pronation with flexion/extension of the elbow prior to symptom onset and consisted of the following: (1) driving with the upper extremity in a prolonged elbow flexion/pronation position; (2) prolonged forcible stretching of the elbow; (3) practicing the violin for several hours a day (severe pain and paresthesia after keeping the bow hand flexed at the elbow with the forearm pronated); and (4) prolonged deep sleep from medication overuse (the hand was in a tightly flexed and pronated position for several hours). Four (8.2%) patients comprised the “other” etiology category. Two of these patients had mass lesions (a cystic lesion and a lipoma) at the volar aspect of the elbow/distal upper arm causing compression of the LACN. The other two did not have a discernable cause and presumably represent entrapment neuropathies. The *p*-value for the etiology distribution was <0.001, indicating a significant variation in causes.

### 3.3. Signs and Symptoms of LACN Neuropathy

**Figure 4:**
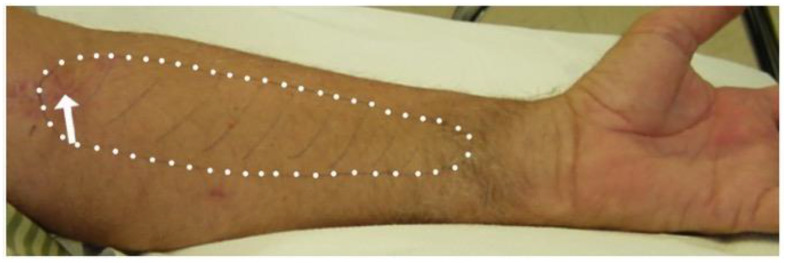
Area of sensory loss (white dots) in a patient with lateral antebrachial cutaneous nerve injury during biceps tendon repair (arrow points to the surgical scar).

At presentation, pain, paresthesia, and/or numbness in the distribution of the LACN were reported in 33 (67.3%), 27 (55.1%), and 23 (46.9%) patients, respectively (Table 1) (Figure 4). The *p*-values for pain were derived from a chi-squared test which assessed whether the proportion of patients experiencing pain (67.3%) was significantly different from 50%. This test yielded a significant result (*p* = 0.015), indicating that most patients with LACN neuropathy experienced pain at a rate significantly higher than 50%. In contrast, the proportions for paresthesia (55.1%) and numbness (46.9%) were not significantly different from 50% (*p*-valutes of 0.190 and 0.553, respectively). Hypoesthesia was detected in 45 (91.8%) patients, with a highly significant *p*-value (<0.001). Dysesthesia was noted in 7 (14.3%), with a *p*-value of 0.052, indicating a marginal association.

### 3.4. Electrodiagnostic Studies

The SNAPs of the LACN on the symptomatic side were absent in 44 (89.8%) patients (Table 1). Of the 5 patients whose SNAPs of the LACN were detected, all had a decreased amplitude, and 2 had an increased latency. The *p*-values for the NCV SNAP categories were all <0.001, indicating highly statistical significance.

### 3.5. NCV SNAP Decreased Amplitude

Table 2 depicts the cohort’s characteristics in relation to the patients with a NCV SNAP with decreased amplitude. A total of 49 patients were analyzed, 44 of whom had no decreased amplitude in NCV SNAP, while 5 patients showed reduced amplitude. The cohort’s median age was 50.0 years [40.0, 56.0]. There was no significant difference in age between patients with and without decreased amplitude (*p* = 0.779). A significantly higher proportion of females were observed in the group with reduced amplitude (100.0% vs. 29.5%, *p* = 0.004). No significant differences were found between the groups in the side affected (*p* = 1.000) or dominant hand (*p* = 1.000). There was also no significant association between NCV SNAP decreased amplitude and symptoms, signs, or manner of the LACN neuropathy.

### 3.6. NCV SNAP Increased Latency

Table 3 highlights the cohort’s characteristics in relation to the patients with a NCV SNAP increased latency. Forty-nine patients were included in the analysis, with forty-seven patients not having increased latency and two patients showing increased latency in NCV SNAP. The cohort’s median age was 50.0 years [40.0, 56.0], and there was no significant difference in age between patients with and without increased latency (*p* = 0.649). A higher proportion of females were present in the group with increased latency (100.0% vs. 34.0%, *p* = 0.130), although this difference was not statistically significant. No significant differences were found in the side affected (*p* = 0.500) or dominant hand (*p* = 1.000) between the groups. There was also no significant association between NCV SNAP increased latency and symptoms, signs, or manner of the LACN neuropathy.

### 3.7. Illustrative Cases

#### 3.7.1. Patient 1

A 57-year-old male complained of numbness of the skin in the left forearm soon after undergoing repair of a ruptured distal biceps tendon by single incision technique under regional block anesthesia. Decreased pinprick and light touch over the volar lateral aspect of the forearm were noted. EDX revealed absent SNAP over the left LACN. The clinical picture suggested iatrogenic injury to the LACN. The symptoms gradually improved over 6 months.

#### 3.7.2. Patient 2

A 28-year-old male complained of paresthesia and pain involving the lateral aspect of the right forearm. The description was a “shock in my right forearm after playing the violin for extended periods of time”. The symptoms were noted primarily when straightening the elbow after keeping it in a flexed and pronated position while holding the bow. A decrease in pinprick sensation over the lateral aspect of the forearm was noted, and no SNAP over the right LACN were detected. Corticosteroid injections in the antecubital fossa lateral to the biceps brachii tendon and limiting the number of hours on the violin led to significant improvement within 2 weeks.

#### 3.7.3. Patient 3

A 40-year-old female underwent a surgical procedure in the proximal forearm to remove a mass which caused pain along the lateral aspect of the forearm and the thumb. Biopsy of the mass revealed a granular cell tumor. The mass was resected along with the brachioradialis muscle into which it had grown. The patient was referred to our Neurodiagnostic Center 6 months postoperatively for numbness of the left forearm and thumb. EDX demonstrated absent SNAP of the left LACN, and the superficial radial nerve confirming nerve injury. The etiology was considered to be iatrogenic injury during tumor excision. The patient did not want to pursue surgical options such as nerve transfer and did not have any follow-up visits.

#### 3.7.4. Patient 4

An 81-year-old female reported a 2-month history of pain and paresthesia of the elbow and lateral aspect of the right forearm and a “painful knot” in the distal upper arm above the elbow. Neurological examination revealed decreased pinprick sensation over the anterolateral aspect of the right forearm and a tender nodular swelling over the distal right upper arm. Tinel sign was positive over the swelling which caused a “shock-like” sensation over the lateral forearm. The SNAP was absent over the right LACN. A cystic lesion was detected by US in the distal lateral upper arm. The patient did not want surgery and was treated with gabapentin for control of paresthesia.

#### 3.7.5. Patient 5

A 37-year-old female complained of painful paresthesia of the lateral aspect of left forearm after venipuncture at the antecubital fossa. The patient experienced severe sharp pain and electric shock-like sensation in the forearm during the procedure. An area of allodynia in the volar lateral aspect of the left forearm was noted. SNAP was absent over the left LACN. Following treatment with gabapentin, the symptoms resolved after 3–4 months without the need for surgical intervention.

## 4. Discussion

### 4.1. Iatrogenic Injuries

Iatrogenic lateral antebrachial cutaneous nerve (LACN) injury has been reported as a complication of distal biceps tendon repair, with the complication rate varying depending upon the surgical technique [7,8,9]. The complication is higher after a single-incision procedure compared to a double-incision technique [7,9]. In Amin and colleagues’ meta-analysis of single-incision versus double-incision surgical techniques for distal biceps tendon repair, LACN neuropraxia was the most common complication in the single-incision group (77 of 785 cases [9.9%]) [7]. LACN neuropraxia occurred in 11 (2.2%) cases in the double-incision group, with a statistically significant difference (<0.001) between the two incision groups. Neuropraxia of the LACN may occur following retraction of the nerve during exposure and preparation of the bicipital tuberosity in the single anterior incision approach [7]. The single-incision method involves retraction of the LACN for a longer period of time than the double-incision approach. In Dunphy and colleagues’ study of 784 surgical repairs of distal biceps tendon ruptures, the most common nerve complication involved the LACN (162 [20.7%] patients), with a significantly higher number following a single-incision repair compared to a double-incision repair (24.4% vs. 4.1%, *p* < 0.001) [9]. In Carroll and colleagues’ study of neurologic complications of distal biceps tendon repair with a single-incision endo button fixation in 50 patients, LACN injury was the most frequent nerve injury [8]. These authors reported additional posterior interosseous nerve (PIN), anterior interosseous nerve (AIN), and superficial radial nerve involvement in 4% of patients [8]. All patients in our series had a single-incision surgery. Of the 11 patients who sustained an iatrogenic injury of the LACN during biceps tendon repair, additional injuries were noted of the superficial radial nerve in 8 (72.3%) patients, PIN in 2 (18.2%), and AIN in 1 (9.1%) by EDX studies. Studies have been conducted to find the optimum trajectory for drilling and placement of the button to avoid injury to the PIN [33].

LACN injuries are known to complicate phlebotomy, either during routine venipuncture or blood donation [4,11,12,13,14,15,34]. Due to the close proximity of the LACN to the cephalic and median cubital veins, it is vulnerable to injury during phlebotomy. LACN injuries are often not recognized and may not be fully reported since the LACN is only a sensory nerve without motor findings [13]. It has been suggested that areas immediately lateral to the biceps tendon and medial to the brachioradialis muscle should be avoided during routine antecubital phlebotomy or be performed superficially in this location [12].

### 4.2. Non-Iatrogenic Injuries

Non-iatrogenic trauma due to laceration injuries of the volar aspect of the forearm accounted for six cases in our study. These injuries occurred in a younger age group compared to the mean age of the entire cohort (32.3 vs. 48.4 years). Compression of the LACN may be result from direct compression or intense physical exertion [16]. Neuropraxia may develop from compression from the biceps brachii during prolonged positional application [16]. The mechanism of LACN injury in this setting involves strenuous elbow extension combined with forearm pronation.

### 4.3. Large Series of LACN Neuropathy in the Literature

Few large series of LACN neuropathy have been reported in the literature (Table 4) [1,4,5]. In Memon and colleagues’ study of 15 patients with LACN neuropathy, a postsurgical etiology was most common (seven patients) during orthopedic surgeries [4]. Two of these patients sustained direct surgical trauma, while five developed symptoms secondary to arm positioning during shoulder (four patients) and knee (one patient) surgeries. Antecubital fossa phlebotomy and intravenous placement were the next most frequent etiologies (four patients). Electrodiagnostic (EDX) studies revealed absent or reduced sensory amplitudes in 13 (86.7%) patients. In Naam and colleagues’ study of 23 patients with LACN neuropathy, 8 (34.8%) sustained elbow trauma, and 17 (73.9%) were Workers’ compensation cases [5]. All had positive nerve conduction study findings consistent with LACN dysfunction, without specific details provided. In Davidson and colleagues’ study of 15 patients with LACN neuropathy, neither the etiology nor EDX findings were reported [1]. The dominant arm was involved in 12 (80%) patients, and 10 (66.7%) had symptoms longer than 6 months in duration. These findings differ from our study that had a lower percentage (57.1%) of patients with dominant arm involvement and a higher percentage (89.8%) with acute symptom onset. Davidson and colleagues described 15 cases of “compression” of the LACN at the bicipital aponeurosis resulting from “strenuous elbow extension or pronation” which is more likely to occur in the dominant hand. Our study comprises much more diverse causes and more than four times the number of patients compared to Davidson et al.’s work which may explain the lower percentage of dominant hand involvement in our study. Our study concurs with that of Memon et al. [4] with respect to iatrogenic injury during orthopedic surgeries as the leading etiology of LACN neuropathy, followed by injuries during phlebotomy. Both studies also reveal sensory nerve action potential (SNAP) abnormalities, with 13 (86.7%) patients with absent or reduced sensory amplitude in the Memon et al. study and absent SNAP in 44 (89.8%) patients in the present study.

Treatment of LACN neuropathy ranges from conservative measures including avoiding the aggravating activity to steroid injections, splinting, and surgical decompression [16]. In Memon and colleagues’ study, 11 patients experienced iatrogenic LACN neuropathy; 7 of these patients had a poor prognosis, 2 had a good recovery, and the prognosis was unknown in 2 patients as they were lost to follow-up [4]. In Stevens and colleagues’ summary of studies in the literature depicting injuries after venous cannulation, eight patients sustained an LACN injury [34]. Of these eight patients, three had a permanent injury, two had a full recovery, and three were lost to follow-up.

### 4.4. Electrodiagnostic Studies

Elbow pain and paresthesia of the forearm are common reasons for referral for EDX studies. The clinical evaluation can provide important clues to the diagnosis, but poor objectivity of the sensory examination can lead to errors. When the paresthesia involves primarily the lateral aspect of the forearm, the differential diagnosis includes a cervical radiculopathy (C5, C6), brachial plexus injury (lateral cord, upper trunk), pronator teres syndrome, biceps tendonitis, radial tunnel syndrome, Parsonage–Turner syndrome, radial and median nerve neuropathies at the level of the elbow, and LACN neuropathy [4,5,27]. EDX studies are often useful in clarifying the correct diagnosis. The prognosis of total loss of SNAP is considered worse than partial loss. The smaller the SNAP, the greater the axon loss causing less favorable outcomes. In nerve injuries, the loss of SNAP signifies axonal injury and less favorable outcomes. Prolonged latency suggests demyelination and a more favorable prognosis.

### 4.5. Ultrasound Studies

**Figure 5:**
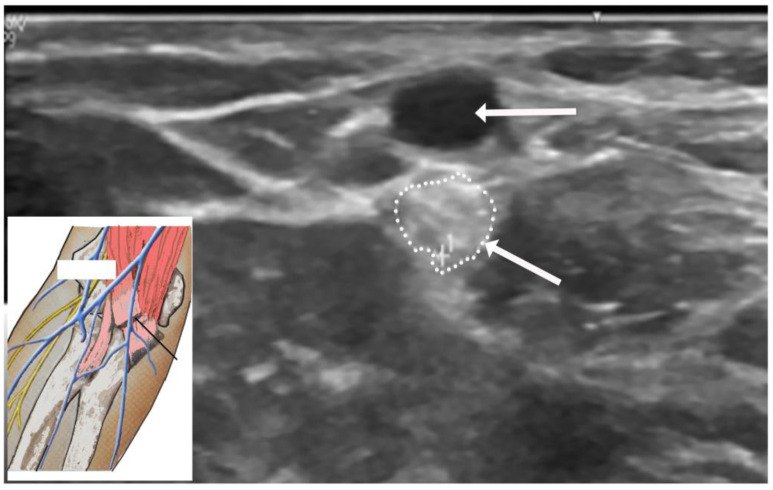
Ultrasound of injury to the lateral antebrachial cutaneous nerve from phlebotomy. Horizontal arrow points to the cephalic vein. Oblique arrow points to enlarged and hyperechoic lateral antebrachial cutaneous nerve. Oblique arrow points to the enlarged and hyperechoic lateral antebrachial cutaneous nerve (encircled by small dots).

US studies may assist in the diagnostic evaluation of LACN neuropathy by differentiating various conditions with symptoms involving the upper arm, elbow, forearm, and wrist [35]. Ultrasound (US) plays an important role in the localization and determination of the etiology of LACN neuropathy. In Chiavaras and colleagues’ study of USs of the LACN with MRI and anatomic correlation in 13 patients with LACN neuropathy, the symptomatic LACN demonstrated fusiform enlargement, increased echogenicity, and loss of the normal fascicular echotexture [35]. The mean cross-sectional area of the symptomatic LACN was 12.0 mm^2^ compared to 3.3 mm^2^ at the same level in the contralateral normal side. While only seven patients in our series underwent an US study, it was valuable in providing details of the masses in the upper extremity compressing the LACN. Figure 5 depicts the US study involving injury to the LACN from phlebotomy.

### 4.6. Strengths and Limitations

The strength of the present study is that it highlights the largest number of patients with LACN neuropathy verified by EDX studies. By determining the etiology of all patients with LACN neuropathy, prophylactic measures can be instituted to avoid injury to the nerve, and the most effective treatment course can be pursued. Limitations include that this study was retrospective and does not have follow-up data after the EDX studies since most patients were evaluated on only one occasion at our Neurodiagnostic Center.

## 5. Conclusions

LACN neuropathy should be considered in the differential diagnosis of patients presenting with pain, paresthesia, or numbness of the forearm, especially after surgical procedures such as biceps tendon repair or activities involving prolonged flexion/extension at the elbow and pronation of the forearm. Without a high index of clinical suspicion and performance of nerve conduction studies of the LACN, such cases may be missed. Protecting the LACN during surgical procedures at the elbow and upper arm may avoid perioperative injury. This paper has the potential to increase the awareness of iatrogenic injury of the LACN during certain surgical procedures such as biceps tendon repair. Our article also aimed to identify and protect the LACN during surgical procedures at the elbow and upper arm to avoid perioperative injury. More frequent use of US studies during difficult phlebotomies may also help to avoid accidental injury to nearby nerves. It may also be useful for patients who are undergoing elbow surgeries to routinely have LACN nerve conduction studies. Sensory nerve intraoperative monitoring may not be easy to alert the surgeon to potential injury during surgery to take remedial measures. If the patient develops sensory symptoms following surgery, nerve conduction studies should be conducted to confirm or rule out nerve injury.

## Figures and Tables

**Table 1:** Demographics and etiologies of lateral antebrachial cutaneous nerve neuropathy at our Neurodiagnostic Center.

Metric		Number of Patients (*n* = 49)	*p*-Value
Age (Mean)		48.4 years (16–81 years)	0.428 *
Gender	Male Female	31 (63.3%) 18 (36.7%)	0.063
Side of symptoms	Left Right	21 (42.9%) 28 (57.1%)	0.317
Dominant hand	Right Left Ambidextrous	43 (87.8%) 3 (6.1%) 3 (6.1%)	<0.001
Symptomatic side corresponds to hand dominance	Yes No	28 (57.1%) 21 (42.9%)	0.317
Symptom onset	Acute Gradual	44 (89.8%) 5 (10.2%)	<0.001
Etiology	Iatrogenic injury (direct or positional)	30 (61.2%)	<0.001
	Biceps tendon repair	11 (36.7%)	
	Phlebotomy	5 (16.7%)	
	Rotator cuff repair	4 (13.3%)	
	Elbow **	2 (6.7%)	
	During intense physical therapy	2 (6.7%)	
	Dupuytren’s contracture	1 (3.3%)	
	Trigger thumb	1 (3.3%)	
	Shoulder (long head of biceps repair)	1 (3.3%)	
	Repair fractured radius/ulna	1 (3.3%)	
	Repair fractured humerus/nerve transfer	1 (3.3%)	
	Granular cell tumor excision/removal of brachioradialis muscle	1 (3.3%)	
	Non-iatrogenic injury	15 (30.6%)	
	Laceration injury	6 (40.0%)	
	Stretch injury ***	5 (33.3%)	
	Fractured humerus	1 (6.7%)	
	Fall (injury of long head of biceps tendon)	1 (6.7%)	
	Workplace injury (biceps tendon tear)	1 (6.7%)	
	Parsonage–Turner syndrome	1 (6.7%)	
	Other	4 (8.2%)	
	Compression by mass (lipoma; cystic lesion)	2 (50.0%)	
	Idiopathic	2 (50.0%)	
Symptoms in distribution of LACN	Pain	33 (67.3%)	0.015
	Paresthesia	27 (55.1%)	0.190
	Numbness	23 (46.9%)	0.553
Signs in distribution of LACN	Hypoesthesia	45 (91.8%)	<0.001
	Dysesthesia	7 (14.3%)	0.052
NCV SNAP	Absent	44 (89.8%)	<0.001
	Decreased amplitude	5 (10.2%)	<0.001
	Increased latency	2 (4.1%)	<0.001

LACN: lateral antebrachial cutaneous nerve; NCV: nerve conduction velocity; SNAP: sensory nerve action potential; * *p*-value from one-sided *t*-test compared to 50 years of age; ** Elbow surgery: reconstructive surgery (1); repair torn ligament (1); *** Stretch injury: driving in prolonged elbow flexion/pronation position (1); prolonged forcible stretching of elbow (1); playing violin (1); grasping a tree with hand (1); upper extremity in “locked up” pronated position for several hours.

**Table 2:** NCV SNAP decreased amplitude.

Characteristics	Overall *n* = 49	No *n* = 44	Yes *n* = 5	*p*-Value
Age	50.0 [40.0, 56.0]	50.0 [42.2, 56.2]	48.0 [37.0, 52.0]	0.779
Female	18 (36.7%)	13 (29.5%)	5 (100.0%)	0.004
Right Side	28 (57.1%)	25 (56.8%)	3 (60.0%)	1.000
Dominant hand				1.000
Left	3 (6.1%)	3 (6.8%)	0 (0.0%)	
Right Side	43 (87.8%)	38 (86.4%)	5 (100.0%)	
Ambidextrous	3 (6.1%)	3 (6.8%)	0 (0.0%)	
Symptomatic Side Corresponds Hand Dominance	28 (57.1%)	25 (56.8%)	3 (60.0%)	1.000
Chronic Onset	5 (10.2%)	3 (6.8%)	2 (40.0%)	0.075
Symptoms (Pain)	33 (67.3%)	30 (68.2%)	3 (60.0%)	1.000
Symptoms (Paresthesia)	28 (57.1%)	23 (52.3%)	5 (100.0%)	0.062
Symptoms (Numbness)	23 (46.9%)	22 (50.0%)	1 (20.0%)	0.353
Signs (Hypoesthesia)	45 (91.8%)	41 (93.2%)	4 (80.0%)	0.359
Signs (Dysesthesia)	7 (14.3%)	6 (13.6%)	1 (20.0%)	0.554
Manner				0.535
Iatrogenic trauma	30 (61.2%)	27 (61.4%)	3 (60.0%)	
Non-iatrogenic trauma	9 (18.4%)	9 (20.5%)	0 (0.0%)	
Other	10 (20.4%)	8 (18.2%)	2 (40.0%)	

**Table 3:** NCV SNAP increased latency.

Characteristics	Overall *n* = 49	No *n* = 47	Yes *n* = 2	*p*-Value
Age	50.0 [40.0, 56.0]	50.0 [41.5, 55.0]	59.0 [48.0, 70.0]	0.649
Female	18 (36.7%)	16 (34.0%)	2 (100.0%)	0.130
Right Side	28 (57.1%)	26 (55.3%)	2 (100.0%)	0.500
Dominant hand				1.000
Left	3 (6.1%)	3 (6.4%)	0 (0.0%)	
Right Side	43 (87.8%)	41 (87.2%)	2 (100.0%)	
Ambidextrous	3 (6.1%)	3 (6.4%)	0 (0.0%)	
Symptomatic Side Corresponds Hand Dominance	28 (57.1%)	26 (55.3%)	2 (100.0%)	0.500
Chronic Onset	5 (10.2%)	3 (6.4%)	2 (100.0%)	0.009
Symptoms (Pain)	33 (67.3%)	32 (68.1%)	1 (50.0%)	1.000
Symptoms (Paresthesia)	28 (57.1%)	26 (55.3%)	2 (100.0%)	0.500
Symptoms (Numbness)	23 (46.9%)	23 (48.9%)	0 (0.0%)	0.491
Signs (Hypoesthesia)	45 (91.8%)	44 (93.6%)	1 (50.0%)	0.158
Signs (Dysesthesia)	7 (14.3%)	6 (12.8%)	1 (50.0%)	0.268
Manner				0.069
Iatrogenic trauma	30 (61.2%)	30 (63.8%)	0 (0.0%)	
Non-iatrogenic trauma	9 (18.4%)	9 (19.1%)	0 (0.0%)	
Other	10 (20.4%)	8 (17.0%)	2 (100.0%)	

**Table 4:** Published series of lateral antebrachial cutaneous nerve neuropathy.

Study	Age (Mean)	Gender	Etiology	EDX Findings
Davidson et al. 1998 [1] (*n* = 15)	18–59 years	M: 10 (66.7%) F: 5 (33.3%)	Not reported	Not reported
Naam et al. 2004 [5] (*n* = 23)	38 years (19–64 years)	M: 15 (65.2%) F: 8 (34.8%)	8: trauma to elbow 17: Workers’ compensation cases	All had positive nerve conduction study findings consistent with LACN dysfunction
Memon et al. 2022 [4] (*n* = 15)	53 years (36–82 years)	M: 7 (46.7%) F: 8 (53.3%)	Iatrogenic injury: 10 (66.7%) (7 during orthopedic surgeries; 3 during antecubital fossa phlebotomy and intravenous placement) Non-iatrogenic injury: 4 (26.7%) (2 from repetitive forearm use, 1 from trauma, and 1 from a dog bite) Other: 1 (6.7%) (idiopathic)	13: absent or reduced sensory amplitude 2: demyelinating pattern with prolonged sensory distal latencies Sensory responses absent in 7/13 patients with an axonal neuropathy pattern
Current study 2024 (*n* = 49)	48.4 years (16–81 years)	M: 31 (63.3%) F: 18 (36.7%)	Iatrogenic injury: 30 (61.2%) (11 during biceps tendon repair, 5 during phlebotomy) Non-iatrogenic injury: 15 (30.6%) (6 due to laceration injury) Other: 4 (8.2%) (2 due to mass compression, 2 idiopathic)	SNAPs absent in 44 (89.8%) patients; SNAPs had a decreased amplitude in 5 (10.2%) patients and an increased latency in 2 (4.1%) patients

EDX: electrodiagnostic; M: male; F: female; LACN: lateral antebrachial cutaneous nerve; SNAP: sensory nerve action potential.

## Data Availability

All of the data for this study are included in the current article.
